# Rasch Analysis of Work-Family Conflict Scale Among Chinese Prison Police

**DOI:** 10.3389/fpsyg.2021.537005

**Published:** 2021-05-03

**Authors:** Wei Chen, Guyin Zhang, Xue Tian, Li Wang, Jie Luo

**Affiliations:** ^1^School of Psychology, Guizhou Normal University, Guiyang, China; ^2^Center for Big Data Research in Psychology, Guizhou Normal University, Guiyang, China; ^3^A Laboratory for Traumatic Stress Studies, CAS Key Laboratory of Mental Health, Institute of Psychology, Beijing, China; ^4^Department of Psychology, University of Chinese Academy of Sciences, Beijing, China

**Keywords:** reliability, validity, differential item functioning, Rasch analysis, work-family conflict, WFCS, prison police

## Abstract

As a special group of police officer, prison police have to endure more work stress and have significant work-family conflict, which may lead to more physical and mental health problems and need to be noticed by the society. The Work-Family Conflict Scale (WFCS) is a brief self-report scale that measures the conflict that an individual experiences between their work and family roles and the extent they interfere with one another. However, there is limited data on the scale’s psychometric properties. The aim of this study was to examine the dimensionality and reliability of the Chinese version of the WFCS. The study sample was made up of a total of 717 Chinese prison police (64.7% male, *M* = 41.73 years, SD = 8.30 years). The Rasch Rating Scale Model (RSM) was used to determine the latent structure and estimate the quality of items and reliability of scale. The principle component analysis (PCA) showed that the assumption of unidimensionality was fulfilled. The infit and outfit mean square (MNSQ) statistics (0.84–1.47) were of a reasonable range, and point-measure correlations (0.64–0.79) indicted good model fit of each item. The item-person separation and reliability indices both met psychometric standards, illustrating good reliability. The person-item map indicated acceptable fit of items and persons, suggesting an alignment between persons and items. In addition, no evidence emerged of differential item functioning across different gender groups. Overall, the WFCS has good reliability and validity, and can be used to accurately evaluate the level of work-family conflict in Chinese prison police.

## Introduction

Police officers overall must bear a higher workload and more risk than those in other occupations, and are more susceptible to the impact of stressful events (Acquadro et al., 2015). These stressors and exposures may increase the risk of suicidal thoughts and behaviors among them (Stanley et al., 2016). Prison police, as a special group of police officer, have to bear more pressures. Their work primarily consists of criminal reform, penalty execution, and prison management. In the process of work, they often need to deal with more threatening emergencies, particularly as it is not uncommon for extreme criminals to focus their dissatisfaction with society onto prison police in hostile and aggressive ways. In addition to their work roles, prison police also hold roles in their family life. When pressure or tasks at work affect prison police in their activities outside work and their ability to fulfill obligations in the family field, work-family conflicts occur (Bhave et al., 2010). These conflicts can negatively affect their mental health and lead to burnout or high turnover rates (Lambert et al., 2016b). To better understand work-family conflict among prison police, this research focuses on the concept of work-family conflict within this field of work by using the Work-Family Conflict Scale (WFCS). Given that Rasch analysis has numerous advantages over Classical Test Theory (CTT), we then use Rasch analysis to test the reliability and validity of the WFCS.

### The Concept of Work-Family Conflict in the Prison Police Context

As a high-risk occupation, police officers may endure unusual threats and physical aggression due to the nature of their work, often suffering from mental health problems because of it (van der Velden et al., 2010). Throughout their career, they will encounter numerous potentially traumatic events, and are exposed to many instances of violence, which together may lead to a higher prevalence in rates of depression and posttraumatic stress disorder (Lawson et al., 2012; Karaffa and Koch, 2016). Police officers in general have also been found to be more susceptible to sleep disorders (Rajaratnam et al., 2011), which further adds to the likelihood of developing more negative emotional states or mental disorders than those in other occupations. As a special group in police, prison police have to face criminals on a daily basis and are more exposed to stressful events because of the danger of criminals. Given these stresses and risks inherent to their career, it is necessary to recognize that the sub-group of prison police are burdened with an intense workload and high pressure on a regular basis. However, in addition to this work pressure, there are many other pressures they also must bear, including family pressures. Given unconventional shift schedules, excessive or intense work-related tasks, and negative job-related emotions, it may hard for prison police to spend much time with their families, and an insufficient amount of time for them to fulfill family obligations. Their work requirements might often cause them to miss important events in their child’s life, which then leads into work-family conflict (Kurtz, 2012). In fact, it has been found that work-family conflict is often cited as being an issue for police (Lambert et al., 2016a). Work-family conflict is understood as being a chronic stressor, and has been associated with impacts on mood, anxiety, less healthy behaviors, poor physical stamina, more self-reported chronic disease, and worse mental health (Moen et al., 2015).

As prison police have an important job shouldering the task of prisoner reform, their mental and physical health directly affects their work efficiency as well as other outcomes. Studies have shown that police officers experience both physiological and psychological stress and burnout, and work-family conflict can be a significant predictor of this (McCarty and Skogan, 2012; Griffin and Sun, 2017). To examine the mental health and subjective well-being of prison police, and therefore find ways to prevent burnout and turnover intention, it is necessary to conduct in-depth research on work-family conflict experienced by prison police.

Work-family conflict refers to the conflict between two areas of work and life, which is usually considered to be in conflict, with tension existing between the different roles in these two areas. Considering the correlation between work and family lives and based on the individual’s subjective experience of incompatible role stress, Kopelman et al. (1983) defined work-family conflict as a conflict between roles, the extent to which the stress an individual feels in one role as being incompatible with the stress of the other. Furthermore, they see stress as incompatible in at least two ways. One is the competition between the demands of different roles for limited time resources, and the other is the pressure associated with one or more roles. In other words, individuals who experience high levels of stress at work may find it difficult to be a good spouse or parents at home. With this definition in mind, research has focused on the extent to which an individual’s work interferes with their family life (i.e., WIF). Different publications have put forward various views on the dimension of work-family conflict. For instance, Greenhaus and Beutell (1985) divided work-family conflict into three types: time-based, strain-based, and behavior-based. They further pointed out that conflict between work and family is bidirectional; conflict caused by work demands is work interference with family (WIF), while family needs become family interference with work (FIW). Integrating previous studies, Gutek et al. (1991) presented six dimensions of work-family conflict, with three different types going in two directions, respectively: time-based WIF, strain-based WIF, behavior-based WIF, time-based FIW, strain-based FIW, and behavior-based FIW. However, it has since been found that individuals experience three times more work-family conflict than family-work conflict (Frone et al., 1992). In other words, work-family interference is a major aspect of work-family conflict experienced by individuals. In addition, as mentioned above, prison police need to spend more time on the job and endure more job-related strain, which is accordant with the conceptual definition of inter-role conflict by Kopelman et al. (1983). For special groups like prison police, the model by Kopelman et al. (1983), compared with the other two bidirectional work-family conflict models, emphasizes more on the subjective feelings of individuals and the interference of work demands on family life. Therefore, we adopt Kopelman’s perspective on work-family conflict and focus on the inter-role conflict that exists between the two roles – work and family life – held by prison police, who feel conflict caused largely by work demands.

### Work-Family Conflict Measurement

Since scholars have different views on the concept and dimensions of work-family conflict, several different scales have been developed to assess the work family conflict. The one compiled by Netemeyer et al. (1996) considered the bidirectional dimension of work-family conflict and contains two factors: work interference WIF and FIW (Netemeyer et al., 1996). However, it has been thought that this two-dimensional scale did not included all three types of pressure proposed by Greenhaus and Beutell (1985), and Carlson et al. (2000) developed another scale with the propose of measuring these three bidirectional types of pressures. This scale contains a total of 18 items with six factors (i.e., time-based WIF, strain-based WIF, behavior-based WIF, time-based FIW, strain-based FIW, and behavior-based FIW, three items for each factor), and was considered a theoretically sound measures of work-family conflict (Matthews et al., 2010). Indeed, this scale was used in many researches and showed good reliability and validity (Carlson et al., 2000; Allen and Armstrong, 2006; Spector et al., 2007; Lim et al., 2011; Annor and Amponsah-Tawiah, 2017). However, there are some limitations to adopt these two scales in this present study. First and foremost, these two scales all focused on the bidirectional dimension of work-family conflict (i.e., WIF and FIW), whereas in prison police samples, the WIF is the main aspect because of they do not have enough time to spend with their families due to the nature of their work, and the pressure of their work is different from that of ordinary people, which makes them less able to cope with their family life. Thus, some of items in these two scales that focus on FIW (e.g., “I have to miss work activities due to the amount of time I must spend on family responsibilities” and “The time I spend with my family often causes me not to spend time in activities at work that could be helpful to my career”) are not fully suitable for the prison police sample. In addition, although the Carlson’s scale is widely used in many studies, researchers also pointed out its limitation (Annor and Amponsah-Tawiah, 2017), more specifically, the survey length (18 items) may limit its usefulness when survey space is at a premium. Given the advantage of reducing assessment time and participant fatigue, a fewer item measure would be chosen to assess behaviors of special staff groups such as prison police. Actually, the WFCS developed by Kopelman et al. (1983) is largely in line with our research needs. This inter-role conflict scale was developed to measure the degree of interference of work-to-family (WIF) from two aspects (e.g., time and pressure). It has one single dimension with a total of eight items, and has been shown to have good reliability (Cronbach’s alpha ranged from 0.87 to 0.89) and validity (negatively related to job satisfaction and family satisfaction, and positively related to depression) (Kopelman et al., 1983; Thomas and Ganster, 1995). One study chose three items of it also have shown acceptable reliability (Cronbach’s alpha = 0.78) using a Maryland and New England nursing sample (Zhang et al., 2016).

Taking into account the characteristics of the specific work-family conflicts that had been already noted among prison police, and due to its simplicity, we decided to use the WFCS developed by Kopelman et al. (1983) for the current study. To our knowledge, far few studies have investigated its measurement performance, which may limit the use of this scale. In addition, considering the special national conditions in China, Chinese prison police are in a state of higher load, intensity and risk, but at the same time they are faced with the reality of low social status and low salary. Studies showed that the overall burnout rate was 51.2% of prison police in China (Yang et al., 2010), and their mental health is lower than that of the general population (Niu et al., 2017), which means it is necessary to pay attention to the work-family conflict of Chinese prison police. However, the WFCS has rarely been used in previous Chinese studies. From a cross-cultural perspective, psychological symptoms can sometimes be expressed differently in different groups of people. This may be due to differences in educational background or in traditional cultures. To determine if there were potential variants in work-family conflict across nations or culture, and verify the applicability and generalization of the scale in this particular group, it seemed prudent to trial the use of the WFCS using a sample of Chinese prison police.

### Rasch Analysis

Rasch analysis has been used in this study to examine the psychometric properties of the WFCS, given that it has advantages over using CTT approaches including confirmatory factor analyses (CFA) (Hagell and Westergren, 2011). Although CTT is still a dominant method of analysis for psychometrics, the theory contains some limitations, such as sample dependent of derived scores (Zile-Tamsen, 2017) and an inability to resolve the limitations of the use of ordinal response scales such as the Likert-type (Medvedev et al., 2018). In CTT, evidence of reliability and validity can also be problematic since that reliability is often proved by Cronbach’s alpha, which is just the degree of inter relevance of items but not an indicator of internal consistency (Sijtsma, 2009). Meanwhile, validity is usually based on the correlation between the score of the scale and other measures, which may or may not be valid by themselves (Zile-Tamsen, 2017). In addition, it is not reasonable to calculate the sum of all the items directly as the overall score considering that each item contributes equally to the total score, because actually different items explain different amounts of information about the underlying structure (Mitchell-Parker et al., 2017). In contrast, Rasch analysis provides a more effective method for measuring psychometric properties of measures. When using Rasch analysis, the ordinal scale scores are translated into an interval metric, and both person and item parameters are able to be compared along the same scale (Mitchell-Parker et al., 2017; Lundgren-Nilsson et al., 2019). Unlike CTT, the measurement error in Rasch analysis does not depend on the specific sample, and it is assumed to be different across different individuals. Furthermore, when assessing the latent structure of the instrument, the Rasch model can easily balance the persons’ level with the items’ level, and it is easier to use to comprehensively evaluate the validity of a scale’s underlying structure (Rustøen et al., 2018).

The Rating Scale Model (RSM) is an extension of the Rasch’s simple logistic model and is used specifically to evaluate scales (Andrich, 1978; Poliandri et al., 2011). It was employed in the current research due to its capacity to include items in Likert-type scale used in this study (Lent and Brown, 2006; Oliveira et al., 2015), and its appropriateness for the expected unidimensional structure of the WFCS. In this study, we firstly verified the factor structure of this scale using a unidimensionality test, which can examine whether the scale satisfies its original assumption of one-dimensionality. In addition, we evaluated the quality of the measure using infit and outfit mean square (MNSQ). Besides, the reliability test was conducted to ensure the consistency of the instrument and person. Furthermore, considering that it is important to determine whether the WFCS demonstrates consistent measurement characteristics across genders, the differential item functioning (DIF) were calculated.

## Materials and Methods

### Participants

A total of 717 prison police in Guiyang, Guizhou Province, China were invited to participate in the study. Subjects ranged from 22 to 55 years of age (*M* = 41.73 years, SD = 8.30 years), 64.66% of them were male and 35.34% were female. A total of 94.70% were married (1.12% single, 3.63% divorced, 0.42% widowed, and 0.14% missing data). Their reported education level was 0.14% high school and below, 97.35% college or university, and 2.37% master or above.

### Measures

To assess work-family conflict we used the Chinese version of the WFCS (Kopelman et al., 1983). This scale requires participants to report the degree of interference of work-to-family that they have experienced. Responses are rated on a five-point Likert-type scale, ranging from 1 (strongly disapprove) to 5 (strongly approve). Higher scores indicate higher levels of conflict. The internal consistency reliability for the study sample was good.

### Procedure

Participants were invited to voluntarily participate by completing the aforementioned self-report questionnaires after authorization from their administrator during their regular working time. The questionnaires also include other demography variables such as gender, age, marital status, and academic degree. The study was approved by the prison administration organization and the Ethics Committee of Guizhou Normal University.

### Data Analysis Strategy

The Rasch RSM was employed using WINSTEPS version 3.74. A principle component analysis (PCA) of the residuals, which relies on unrotated solutions, was used to examine the unidimensionality (Williams et al., 2012). Unlike traditional factor analyses, the PCA of the residuals is conducted after excluding the target construct, meanwhile, it detects secondary dimensions. The unidimensionality test allows us to identify the unique principal factor of the instrument. The assumption of unidimensionality can be supported when an eigenvalue for the first contrast of residual is lower than 2.0, or the proportion of variance explained by the measure is ≥20%.

The model fit was examined using infit and outfit MNSQ for item and person parameters to show whether each item fit model expectations. Good fit occurs when there is no significant difference between the model and the observed response pattern. Items with insufficient model fit means that they should be dropped from subsequent analyses. Values ranging from 0.5 to 1.5 indicate acceptable model fit (Pichardo et al., 2018), and the point-measure correlations were calculated to provide evidence of consistency between the score of each item and the overall latent trait.

The person reliability index (PRI) and item reliability index (IRI), which interpreted similar to Cronbach’s alpha, were used to indicate the consistency of the instrument and person; a value of >0.7 indicates acceptable model fit. The person separation index (PSI) and item separation index (ISI) were used to estimate the spread of items and persons, that is, to verify whether the instrument was sensitive enough to distinguish different levels of sample in this study; a value of >2.5 indicates good model fit (Tennant and Conaghan, 2007).

Person-item map was used to illustrate how, for each respondent, the item difficulties match the latent trait, and to assess whether items had floor or ceiling effects. In this model, the acceptable results are an alignment between items and persons, and a normal distribution of persons. Item difficulty was depicted on the right of the vertical axis and individual trait were on the left (Ningrum et al., 2019).

To estimate if person with the same attribute level would have the same respond probability to an item of the WFCS across different genders, differential item functioning (DIF) based on ordinal logistic regression was examined. DIF can be divided into uniform and non-uniform DIF; the former is analogous to effect modification and the latter is analogous to confounding (Crane et al., 2006). No evidence of DIF means that the scale has the same meaning and function across different genders. By testing the significance of the regression coefficient, we were able to determine whether there is group bias in each item.

## Results

### Unidimensional

As shown in Table 1, the PCA of the residuals produced an Eigenvalues of 10.0, and the proportion of variance explained by the measure is 55.6%, much higher than the acceptable minimum value of 20%. At the same time, the first contrast of residual has a value of lower than 2.0.

**TABLE 1:** Variance of standardized residuals.

	Eigenvalues	Observed	Expected
		(%)	(%)
**Goal setting**			
Total raw variance	18.0	100	100
Raw variance explained by measures	10.0	55.6	55.5
Raw variance explained by persons	5.9	32.7	32.6
Raw variance explained by items	4.1	22.9	22.9
Raw unexplained variance (total)	8.0	44.4	44.5
Raw variance unexplained in first contrast	1.6	8.9	20.1

### Model Fit of Items

The infit and outfit MNSQ values ranged from 0.84 to 1.47, indicating all items show acceptable fit to the model, and no need to be dropped from subsequent analyses. Likewise, in the PT-Measure-Correlation column, good fitting items showed positive correlations. The correlation values tend to be high, ranging from 0.64 to 0.79, and are all close to the expectations of the model (see Table 2).

**TABLE 2:** Model fit for each item.

Item	Model SE	MNSQ		PT-measure	
		Infit	Outfit	Correlation	Expected
1	0.05	1.17	1.22	0.71	0.74
2	0.05	0.89	0.89	0.76	0.74
3	0.05	0.86	0.89	0.76	0.74
4	0.05	1.36	1.47	0.64	0.74
5	0.05	1.09	1.12	0.74	0.75
6	0.05	0.87	0.85	0.77	0.74
7	0.05	0.87	0.84	0.77	0.73
8	0.05	0.86	0.85	0.79	0.74

### Reliability

The reliability and separation index values for person and item are presented in Table 3. Both IRI and PRI show high values, indicating that either item and response difficulties or the latent score for persons were all consistent. In addition, the ISI and PSI values are more than the criterion (2.5), indicating good item and person discrimination.

**TABLE 3:** Item and person reliability.

	Reliability index	Separation index
Item	0.92	3.35
Person	0.87	2.53

### Person-Item Map

**FIGURE 1:**
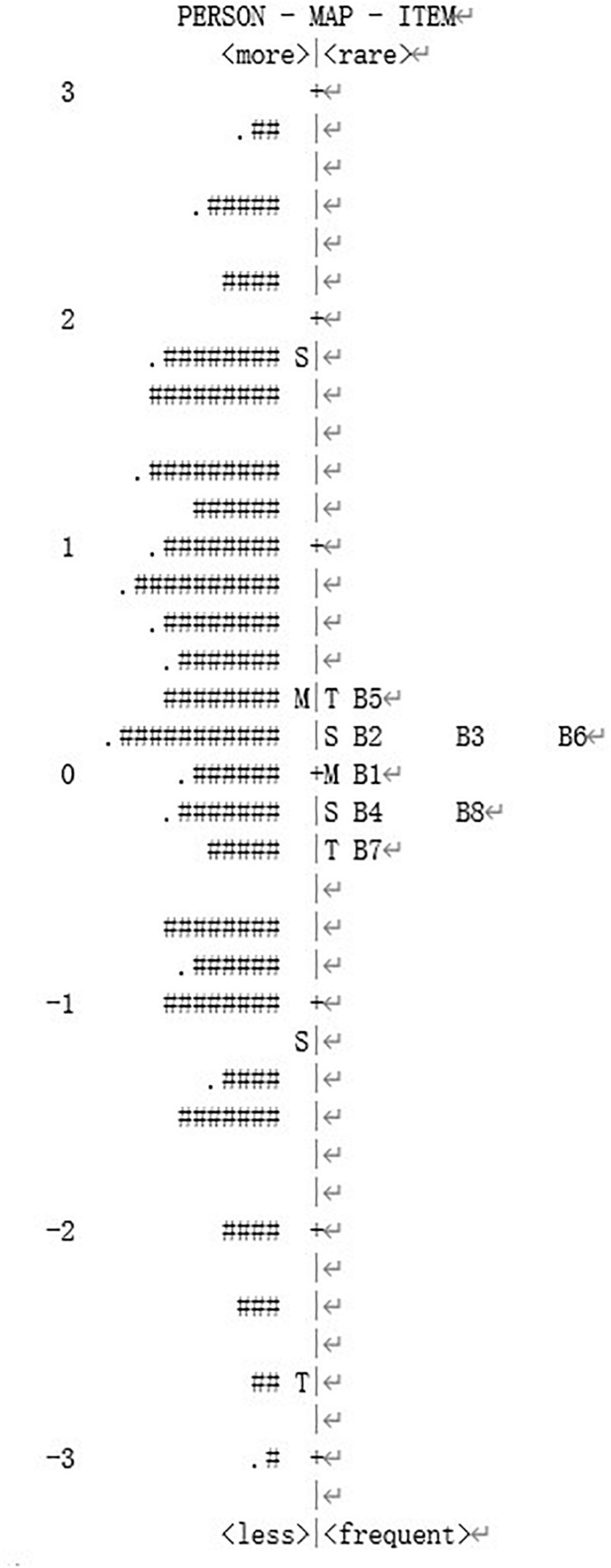
Person-item map for the WFCS.

The distributions of items and persons were plotted in this logit ruler, the former is on the right side of the map, the latter on the left. High-trait persons are located in the upper part, low-trait persons below, and the distribution of items are in the same. As shown in Figure 1, the latent trait of individuals achieved normal distribution. The mean item difficulty was 0.00, and the mean person latent trait was 0.50. The difference between them was no greater than 0.5 logits, indicating that the result was acceptable (Bond and Fox, 2015). In other words, the item difficulty can properly distinguish the differences of the person latent trait. However, it is worth noting that there were still many people who possess the latent trait outside the range (−1, +1), which means that these items were insufficient to cover the individuals’ range of ability. Overall, individuals with higher work-family conflict were more likely to choose the high response category, and the WFCS may be more sensitive for individuals with medium latent trait.

### Differential Item Functioning

A DIF based on an ordinal logistic regression model was run to test differences in the item parameters for gender groups. As seen in Table 4, no evidence of uniform DIF or non-uniform DIF was found between males and females for any items, thus indicating that there was no item bias in the WFCS between genders.

**TABLE 4:** Differential item functioning (DIF) for gender.

	Uniform DIF		Non-uniform DIF	
Item	*Change in estimation*	DIF	*P[Dif(LL)]*	DIF
1	−0.01	No	0.38	No
2	0.02	No	0.43	No
3	0.01	No	0.22	No
4	0.02	No	0.52	No
5	0.01	No	0.44	No
6	0.00	No	0.86	No
7	−0.01	No	0.62	No
8	−0.01	No	0.44	No

*Uniform DIF if change in estimation > 0.1, non-uniform DIF if p-value of the difference of likelihood-ratio < 0.05.*

## Dicussion

Work-family conflict is a strong source of stress for workers and can affect their work, family, and health (Zhang et al., 2016). Researches have shown that being a police officer in general is particularly stressful compared to other occupations. Police often fall back to negative coping strategies, sometimes choosing suicide or suicide intention to deal with stress in extreme cases (Burke, 1993; Griffin and Sun, 2017). Given that prison police are a special group of police under particularly unique pressure, it is necessary to pay attention to the work-family conflict they experience. Considering the fit of the concept definition to the characteristics of work-family conflict of prison police in particular, we selected the WFCS as developed by Kopelman et al. (1983) to test its measurement properties using a sample of Chinese prison police.

In terms of analysis, CTT has several limitations, such as a tendency to bias toward central scores and the sample-dependence of derived scores. However, using Rasch analysis it is possible to transform ordinal scale scores into an interval metric, and then place the difficulty level of items and the individual level of persons into the same logit ruler (Wright and Linacre, 1989; Hendriks et al., 2012). This makes Rasch analysis an effective method to explore the psychometric properties of the WFCS measure and assess the response bias.

The primary assumption of the RSM is unidimensionality. The PCA of the residuals examined whether the first contrast of residual value or the explained variance proportion indicated fulfillment of the assumption. Results showed that the WFCS demonstrated good unidimensionality for the Chinese prison police sample, and is consistent with the one-factor structure of the original scale (Kopelman et al., 1983).

According to the infit and outfit MNSQ and the PT-Measure-Correlation values, a high agreement between items and model was found. This means in the data, there were no unexpected patterns, and all the items of the WFCS fit the RSM model well. It is worth noting that the infit and outfit MNSQ values can also be used as evidence for the verification of unidimensionality (Brentari and Golia, 2007). This data shows the same support for the one-factor structure as the PCA result, indicating that the WFCS is a good one-dimensional measurement.

Sufficient results of the PSI, ISI, PRI, and IRI were also obtained. The PRI and IRI, which are similar to the Cronbach’s alpha, were 0.87 and 0.92, respectively, showing high reliability values for persons and items, namely that consistency was gained either in the rank order of item-response difficulties or in the rank order of individual’s level of WFC. The minimum acceptable criteria of PSI and ISI is 1.5 (Tennant and Conaghan, 2007), but in this current study, these values were both higher than 2.5, more than satisfying this criteria. This suggests good item discrimination between individuals with high and low levels of WFC, and good person discrimination in different items.

The item distribution of the scale basically matched the distribution of the latent trait level of the persons in the Wright Map. The difference between the mean item location and the mean person location is acceptable, which indicates that the WFCS is adequately suited for measuring WFC in this sample, as well as of the results of the PRI and IRI. Additionally, the distribution of latent trait level was achieved normal distribution, demonstrating that the items can effectively distinguish the latent trait of persons. But it is worth noting that a fraction of participants chosen the highest choice for some of the items, suggesting that the WFCS may be a more precise measure of medium outcome expectations.

To ensure the evaluation of validity, it is important that the scale is not biased toward any demographic characteristic (Rustøen et al., 2018). In the test of measurement equivalence, there was neither uniform DIF (*p*-value from −0.01 to 0.02) nor non-uniform DIF (*p*-value from 0.22 to 0.86), suggesting that the WFCS is equally effective across genders and there is no interference of demographic characteristic.

Examination of the structure of the measure and of its psychometric properties using Rasch analysis indicated that the unidimensionality assumption of the WFCS was fulfilled and that the reliability presented good results. Altogether, then, it can be concluded that the WFCS can be an effective instrument to evaluate work-family conflict in Chinese prison police.

## Limitations

This study has some limitations. First, all participants were sampled from the same geographic area, which might have affected variability. Future studies should attempt to draw participants from different areas. In addition, due to the detection of psychometric properties is different in CTT and Rasch, the present study did not include the validity analysis of the WFCS. Future studies should further detect the validity of this scale. Also, because of the particularity of prison police work, males dominated the sample used. Therefore, future research should explore work-family conflict with a focus on female prison police and examine the effects of any gender gap.

## Data Availability Statement

The datasets generated for this study are available on request to the corresponding author.

## Ethics Statement

The studies involving human participants were reviewed and approved by the Human Subjects Review Committee at Guizhou Normal University. Written informed consent for participation was not required for this study in accordance with the national legislation and the institutional requirements.

## Author Contributions

WC, GZ, and XT made main contribution to this study, including come up with ideal, plan, execution, and provide data analysis strategies of this study, and draft of the manuscript. LW and JL provided suggestions on revision and confirmed the final version to be published. All authors of this research article have participated in the discussion of theoretical framework and content modification.

## Conflict of Interest

The authors declare that the research was conducted in the absence of any commercial or financial relationships that could be construed as a potential conflict of interest.
